# Program Synthesis Using Deduction-Guided Reinforcement Learning

**DOI:** 10.1007/978-3-030-53291-8_30

**Published:** 2020-06-16

**Authors:** Yanju Chen, Chenglong Wang, Osbert Bastani, Isil Dillig, Yu Feng

**Affiliations:** 8 Microsoft Research Lab, Redmond, WA USA; 9 University of Southern California, Los Angeles, CA USA; 10 University of California, Santa Barbara, Santa Barbara, CA 93106 USA; 11 University of Washington, Seattle, WA 98115 USA; 12 University of Pennsylvania, Philadelphia, PA 19104 USA; 13 The University of Texas at Austin, Austin, TX 78712 USA

## Abstract

In this paper, we present a new program synthesis algorithm based on reinforcement learning. Given an initial policy (i.e. statistical model) trained off-line, our method uses this policy to guide its search and gradually improves it by leveraging feedback obtained from a deductive reasoning engine. Specifically, we formulate program synthesis as a reinforcement learning problem and propose a new variant of the *policy gradient* algorithm that can incorporate feedback from a deduction engine into the underlying statistical model. The benefit of this approach is two-fold: First, it combines the power of deductive and statistical reasoning in a unified framework. Second, it leverages deduction not only to *prune* the search space but also to *guide* search. We have implemented the proposed approach in a tool called Concord and experimentally evaluate it on synthesis tasks studied in prior work. Our comparison against several baselines and two existing synthesis tools shows the advantages of our proposed approach. In particular, Concord solves 15% more benchmarks compared to Neo, a state-of-the-art synthesis tool, while improving synthesis time by 8.71$\times$ on benchmarks that can be solved by both tools.

## Introduction

Due to its potential to significantly improve both programmer productivity and software correctness, *automated program synthesis* has gained enormous popularity over the last decade. Given a high-level specification of user intent, most modern synthesizers perform some form of backtracking search in order to find a program that satisfies the specification. However, due to the enormous size of the search space, synthesizers additionally use at least one of two other techniques, namely deduction and statistical reasoning, to make this approach practical. For example, many recent synthesis techniques use lightweight program analysis or logical reasoning to significantly prune the search space [18, 19, 39, 53]. On the other hand, several recent approaches utilize a statistical model (trained off-line) to bias the search towards programs that are more likely to satisfy the specification [2, 4, 7, 19]. While both deductive and statistical reasoning have been shown to dramatically improve synthesis efficiency, a key limitation of existing approaches is that they do not tightly combine these two modes of reasoning. In particular, although logical reasoning often provides very useful feedback at synthesis time, existing synthesis algorithms do not leverage such feedback to improve their statistical model.

In this paper, we propose a new synthesis algorithm that meaningfully combines deductive and statistical reasoning. Similar to prior techniques, our approach starts with a statistical model (henceforth called a *policy*) that is trained off-line on a representative set of training problems and uses this policy to guide search. However, unlike prior techniques, our method *updates* this policy on-line at synthesis time and gradually improves the policy by incorporating feedback from a deduction engine.

To achieve this tight coupling between deductive and statistical reasoning, we formulate syntax-guided synthesis as a reinforcement learning (RL) problem. Specifically, given a context-free grammar for the underlying DSL, we think of partial (i.e., incomplete) programs in this DSL as states in a Markov Decision Process (MDP) and actions as grammar productions. Thus, a *policy* of this MDP specifies how a partial program should be extended to obtain a more specific program. Then, the goal of our reinforcement learning problem is to improve this policy over time as some partial programs are proven infeasible by an underlying deduction engine.

**Fig. 1.**
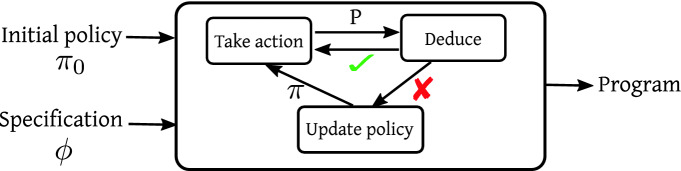
Overview of our synthesis algorithm

While the framework of reinforcement learning is a good fit for our problem, standard RL algorithms (e.g., policy gradient) typically update the policy based on feedback received from states that have *already* been explored. However, in the context of program synthesis, deductive reasoning can also provide feedback about states that have *not* been explored. For example, given a partial program that is infeasible, one can analyze the root cause of failure to infer *other* infeasible programs [18, 54]. To deal with this difficulty, we propose an *off-policy* reinforcement learning algorithm that can improve the policy based on such additional feedback from the deduction engine.

As shown schematically in Fig. 1, our synthesis algorithm consists of three conceptual elements, indicated as “Take action”, “Deduce”, and “Update policy”. Given the current policy $\pi$ and partial program *P*, “Take action” uses $\pi$ to expand *P* into a more complete program $P'$. Then, “Deduce” employs existing deductive reasoning techniques (e.g., [18, 32]) to check whether $P'$ is feasible with respect to the specification. If this is not the case, “Update policy” uses the feedback provided by the deduction engine to improve $\pi$. Specifically, the policy is updated using an off-policy variant of the *policy gradient* algorithm, where the gradient computation is adapted to our unique setting.

We have implemented the proposed method in a new synthesis tool called Concord and empirically evaluate it on synthesis tasks used in prior work [2, 18]. We also compare our method with several relevant baselines as well as two existing synthesis tools. Notably, our evaluation shows that Concord can solve 15% more benchmarks compared to Neo (a state-of-the-art synthesis tool), while being 8.71$\times$ faster on benchmarks that can be solved by both tools. Furthermore, our ablation study demonstrates the empirical benefits of our proposed reinforcement learning algorithm.

To summarize, this paper makes the following key contributions:

- We propose a new synthesis algorithm based on reinforcement learning that tightly couples statistical and deductive reasoning.
- We describe an off-policy reinforcement learning technique that uses the output of the deduction engine to gradually improve its policy.
- We implement our approach in a tool called Concord and empirically demonstrate its benefits compared to other state-of-the-art tools as well as ablations of our own system.

The rest of this paper is structured as follows. First, we provide some background on reinforcement learning and MDPs (Sect. 2) and introduce our problem formulation in Sect. 3. After formulating the synthesis problem as an MDP in Sect. 4, we then present our synthesis algorithm in Sect. 5. Sections 6 and 7 describe our implementation and evaluation respectively. Finally, we discuss related work and future research directions in Sect. 8 and 9.

## Background on Reinforcement Learning

At a high level, the goal of reinforcement learning (RL) is to train an agent, such as a robot, to make a sequence of decisions (e.g., move up/down/left/right) in order to accomplish a task. All relevant information about the environment and the task is specified as a *Markov decision process (MDP)*. Given an MDP, the goal is to compute a policy that specifies how the agent should act in each state to maximize their chances of accomplishing the task.

In the remainder of this section, we provide background on MDPs and describe the policy gradient algorithm that our method will build upon.

***Markov Decision Process.*** We formalize a *Markov decision process (MDP)* as a tuple $\mathcal {M}=(\mathcal {S},\mathcal {S}_I,\mathcal {S}_T,\mathcal {A},\mathcal {F},\mathcal {R})$, where:

- $\mathcal {S}$ is a set of *states* (e.g., the robot’s current position),
- $\mathcal {S}_I$ is the initial state distribution,
- $\mathcal {S}_T$ is a set of the final states (e.g., a dead end),
- $\mathcal {A}$ is a set of actions (e.g., move up/down/left/right),
- $\mathcal {F}:\mathcal {S}\times \mathcal {A}\rightarrow \mathcal {S}$ is a set of transitions,
- $\mathcal {R}:\mathcal {S}\rightarrow \mathbb {R}$ is a reward function that assigns a reward to each state (e.g., 1 for reaching the goal and 0 otherwise).

In general, transitions in an MDP can be stochastic; however, for our setting, we only consider deterministic transitions and rewards.

***Policy.*** A policy for an MDP specifies how the agent should act in each state. Specifically, we consider a (stochastic) *policy* $\pi :\mathcal {S}\times \mathcal {A}\rightarrow \mathbb {R}$, where $\pi (S,A)$ is the probability of taking action *A* in state *S*. Alternatively, we can also think of $\pi$ as a mapping from states to distributions over actions. Thus, we write $A \sim \pi (S)$ to denote that action *A* is sampled from the distribution for state *s*.

***Rollout.*** Given an MDP $\mathcal {M}$ and policy $\pi$, a *rollout* is a sequence of state-action-reward tuples obtained by sampling an initial state and then using $\pi$ to make decisions until a final state is reached. More formally, for a rollout of the form:

$$\begin{aligned} \zeta&=((S_1,A_1,R_1),...,(S_{m-1},A_{m-1},R_{m-1}),(S_m,\varnothing ,R_m)), \end{aligned}$$

we have $S_m \in \mathcal {S}_T$, $S_1\sim \mathcal {S}_I$ (i.e., $S_1$ is sampled from an initial state), and, for each $i\in \{1,...,m-1\}$, $A_i \sim \pi (S_i)$, $R_i=\mathcal {R}(S_i)$, and $S_{i+1}=\mathcal {F}(S_i,A_i)$.

In general, a policy $\pi$ induces a distribution $\mathcal {D}_{\pi }$ over the rollouts of an MDP $\mathcal {M}$. Since we assume that MDP transitions are deterministic, we have:

$$\begin{aligned} \mathcal {D}_{\pi }(\zeta )=\prod _{i=1}^{m-1}\pi (S_i,A_i). \end{aligned}$$

***RL Problem.*** Given an MDP $\mathcal {M}$, the goal of reinforcement learning is to compute an *optimal* policy $\pi ^*$ for $\mathcal {M}$. More formally, $\pi ^*$ should maximize *cumulative expected reward*:

$$\begin{aligned} \pi ^*={\mathop {\hbox {arg max}}\limits _\pi }\, J(\pi )  \end{aligned}$$

where the *cumulative expected reward* $J(\pi )$ is computed as follows:

$$ J(\pi )=\mathbb {E}_{\zeta \sim \mathcal {D}_{\pi }}\left[ \sum _{i=1}^mR_i\right] $$

***Policy Gradient Algorithm.*** The *policy gradient algorithm* is a well-known RL algorithm for finding optimal policies. It assumes a parametric policy family $\pi _{\theta }$ with parameters $\theta \in \mathbb {R}^d$. For example, $\pi _{\theta }$ may be a deep neural network (DNN), where $\theta$ denotes the parameters of the DNN. At a high level, the policy gradient algorithm uses the following theorem to optimize $J(\pi _{\theta })$ [48]:

### Theorem 1

We have

1 $$\begin{aligned} \nabla _{\theta }J(\pi _{\theta })=\mathbb {E}_{\zeta \sim \mathcal {D}_{\pi _{\theta }}}[\ell (\zeta )] \qquad \text {where}\qquad \ell (\zeta )=\sum _{i=1}^{m-1}\left( \sum _{j=i+1}^mR_j\right) \nabla _{\theta }\log \pi _{\theta }(S_i,A_i). \end{aligned}$$

In this theorem, the term $\nabla _{\theta }\log \pi _{\theta }(S_i,A_i)$ intuitively gives a direction in the parameter space that, when moving the policy parameters towards it, increases the probability of taking action $A_i$ at state $S_i$. Also, the sum $\sum _{j = i+1}^m R_j$ is the total future reward after taking action $A_i$. Thus, $\ell (\zeta )$ is just the sum of different directions in the parameter space weighted by their corresponding future reward. Thus, the gradient $\nabla _\theta J(\pi _\theta )$ moves policy parameters in a direction that increases the probability of taking actions that lead to higher rewards.

Based on this theorem, we can estimate the gradient $\nabla _{\theta }J(\pi _{\theta })$ using rollouts sampled from $\mathcal {D}_{\pi _{\theta }}$:

2 $$\begin{aligned} \nabla _{\theta }J(\pi _{\theta })\approx \frac{1}{n}\sum _{k=1}^n\ell (\zeta ^{(k)}), \end{aligned}$$

where $\zeta ^{(k)}\sim \mathcal {D}_{\pi _{\theta }}$ for each $k\in \{1,...,n\}$. The policy gradient algorithm uses stochastic gradient ascent in conjunction with Eq. (2) to maximize $J(\pi _{\theta })$ [48].

## Problem Formulation

  In this paper, we focus on the setting of syntax-guided synthesis [1]. Specifically, given a domain-specific language (DSL) *L* and a specification $\phi$, our goal is to find a program in *L* that satisfies $\phi$. In the remainder of this section, we formally define our synthesis problem and clarify our assumptions.

***DSL.*** We assume a domain-specific language *L* specified as a context-free grammar $L=(V,\varSigma ,R,S)$, where $V, \varSigma$ denote non-terminals and terminals respectively, *R* is a set of productions, and *S* is the start symbol.

### Definition 1

**(Partial program).** A *partial program* *P* is a sequence $P\in (\varSigma \cup V)^*$ such that $S \overset{*}{\Rightarrow }P$ (i.e., *P* can be derived from *S* via a sequence of productions). We refer to any non-terminal in *P* as a hole *hole*, and we say that *P* is *complete* if it does not contain any holes.

Given a partial program *P* containing a hole *H*, we can fill this hole by replacing *H* with the right-hand-side of any grammar production *r* of the form $H \rightarrow e$. We use the notation $P\overset{r}{\Rightarrow }P'$ to indicate that $P'$ is the partial program obtained by replacing the first occurrence of *H* with the right-hand-side of *r*, and we write Fill$(P, r) = P'$ whenever $P \overset{r}{\Rightarrow } P'$.

**Fig. 2.**
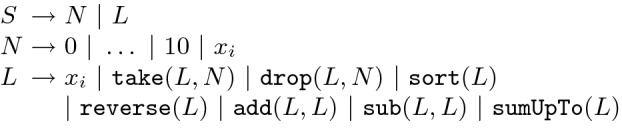
A simple programming language used for illustration. Here, take (resp. drop) keeps (resp. removes) the first *N* elements in the input list. Also, add (resp. sub) compute a new list by adding (resp. subtracting) elements from the two lists pair-wise. Finally, sumUpTo generates a new list where the *i*’th element in the output list is the sum of all previous elements (including the *i*’th element) in the input list.

### Example 1

Consider the small programming language shown in Fig. 2 for manipulating lists of integers. The following partial program *P* over this DSL contains three holes, namely $L_1, L_2, N_1$:

$$ \mathtt{add}(L_1, \mathtt{take}(L_2, N_1)) $$

Now, consider the production $r \equiv L \rightarrow \mathtt{reverse}(L)$. In this case, Fill(*P*, *r*) yields the following partial program $P'$:

$$ \mathtt{add}(\mathtt{reverse}(L_1), \mathtt{take}(L_2, N_1)) $$

***Program Synthesis Problem.*** Given a specification $\phi$ and language $L=(V,\varSigma ,R,S)$, the goal of program synthesis is to find a *complete* program *P* such that $S \overset{*}{\Rightarrow } P$ and *P* satisfies $\phi$. We use the notation $P\models \phi$ to indicate that *P* is a complete program that satisfies specification $\phi$.

***Deduction Engine.*** In the remainder of this paper, we assume access to a *deduction engine* that can determine whether a partial program *P* is *feasible* with respect to specification $\phi$. To make this more precise, we introduce the following notion of feasibility.

### Definition 2

**(Feasible partial program).** Given a specification $\phi$ and language $L=(V,\varSigma ,R,S)$, a partial program *P* is said to be *feasible* with respect to $\phi$ if there exists any complete program $P'$ such that $P \overset{*}{\Rightarrow } P'$ and $P' \models \phi$.

In other words, a feasible partial program can be refined into a complete program that satisfies the specification. We assume that our deduction oracle over-approximates feasibility. That is, if *P* is feasible with respect to specification $\phi$, then Deduce($P,\,\phi$) should report that *P* is feasible but not necessarily vice versa. Note that almost all deduction techniques used in the program synthesis literature satisfy this assumption [18, 19, 21, 27, 53].

### Example 2

Consider again the DSL from Fig. 2 and the specification $\phi$ defined by the following input-output example:

$$ [65, 2, 73, 62, 78] \mapsto [143, 129, 213, 204, 345] $$

The partial program $\mathtt{add}(\mathtt{reverse}(x), \mathtt{take}(x, N))$ is infeasible because, no matter what production we use to fill non-terminal *N*, the resulting program cannot satisfy the provided specification for the following reason:

- Given a list *l* and integer *n* where $n < {length}(l)$, take(*l*, *n*) returns the first *n* elements in *l*. Thus, the length of take(*l*, *n*) is smaller than that of *l*.
- The construct reverse(*l*) reverses its input; thus, the size of the output list is the same as its input.
- Finally, add($l_1, l_2$) constructs a new list by adding the elements of its input lists pair-wise. Thus, add expects the two input lists to be the same size.
- Since the outputs of reverse and take do not have the same size, we cannot combine them using add.

Several techniques from prior work (e.g., [18, 19, 39, 53]) can prove the infeasibility of such partial programs by using an SMT solver (provided specifications are given for the DSL constructs).

Beyond checking feasibility, some deduction techniques used for synthesis can also provide additional information [18, 32, 54]. In particular, given a partial program *P* that is infeasible with respect to specification $\phi$, several deduction engines can generate a set of other infeasible partial programs $P_1, \ldots , P_n$ that are infeasible for the same reason as *P*. To unify both types of feedback, we assume that the output of the deduction oracle $\mathcal {O}$ is a set *S* of partial programs such that *S* is empty if and only if $\mathcal {O}$ decides that the partial program is feasible.

This discussion is summarized by the following definition:

### Definition 3

**(Deduction engine).** Given a partial program *P* and specification $\phi$, Deduce($P,\,\phi$) yields a set of partial programs *S* such that (1) if $S \ne \varnothing$, then *P* is infeasible, and (2) for every $P' \in S$, it must be the case that $P'$ is infeasible with respect to $\phi$.

### Example 3

Consider again the same infeasible partial program *P* given in Example 2. Since drop(*l*, *n*) drops the first *n* elements from list *l* (where $n < length (l))$, it also produces a list whose length is smaller than that of the input. Thus, the following partial program $P'$ is also infeasible for the same reason as *P*:

$$ P' \equiv \mathtt{add}(\mathtt{reverse}(x), \mathtt{drop}(x, N)) $$

Thus, Deduce($P,\,\phi$) may return the set $\{ P, P'\}$.

## MDP Formulation of Deduction-Guided Synthesis

Given a specification $\phi$ and language $L=(V,\varSigma ,R,S)$, we can formulate the program synthesis problem as an MDP $\mathcal {M}_{\phi } = (\mathcal {S},\mathcal {S}_I,\mathcal {S}_T,\mathcal {A},\mathcal {F},\mathcal {R})$, where:

- States $\mathcal {S}$ include all partial programs *P* such that $S \overset{*}{\Rightarrow } P$ as well as a special label $\bot$ indicating a syntactically ill-formed partial program
- $\mathcal {S}_I$ places all probability mass on the empty program *S*, i.e.,
  $$ \mathcal {S}_I(P) = \left\{ \begin{array}{ll} 1 &{} \text {if } P = S\\ 0 &{} \text {if } P \ne S \end{array} \right. $$
- $\mathcal {S}_T$ includes complete programs as well as infeasible partial programs, i.e.,
  $$ P \in \mathcal {S}_T \iff \textsc {IsComplete}(P) \ \vee \ \textsc {Deduce}(P, \phi ) \ne \varnothing \vee P = \bot $$
- Actions $\mathcal {A}$ are exactly the productions *R* for the DSL
- Transitions $\mathcal {F}$ correspond to filling a hole using some production i.e.,
  $$ \mathcal {F}(P, \ r = (H \rightarrow e)) = \left\{ \begin{array}{ll} \bot &{} \text {if } H \text { is not a hole in } P \\ \textsc {Fill}(P, r) \ \ \ &{} \text {otherwise} \end{array} \right. $$
- The reward function penalizes infeasible programs and rewards correct solutions, i.e.,
  $$\begin{aligned} \mathcal {R}(P)= {\left\{ \begin{array}{ll} 1&{}\text {if } P\models \phi \\ -1&{}\text {if } P = \bot \vee \textsc {Deduce}(P,\phi )\ne \varnothing \vee (\textsc {IsComplete}(P) \wedge P \not \models \phi )\\ 0&{}\text {otherwise}. \end{array}\right. } \end{aligned}$$

Observe that our reward function encodes the goal of synthesizing a complete program *P* that satisfies $\phi$, while avoiding the exploration of as many infeasible programs as possible. Thus, if we have a good policy $\pi$ for this MDP, then a rollout of $\pi$ is likely to correspond to a solution of the given synthesis problem.

### Example 4

Consider the same specification (i.e., input-output example) $\phi$ from Example 2 and the DSL from Example 1. The partial program

$$ P \equiv \mathtt{add}(\mathtt{reverse}(x), \mathtt{take}(x, N)) $$

is a terminal state of $\mathcal {M}_\phi$ since Deduce($P,\,\phi$) yields a non-empty set, and we have $\mathcal {R}(P) = -1$. Thus, the following sequence corresponds to a rollout of $\mathcal {M}_\phi$:

$$ \begin{array}{l} (S, S \rightarrow L, 0), \ (L, L \rightarrow \mathtt{add}(L, L), 0), \ (\mathtt{add}(L_1, L_2), L \rightarrow \mathtt{reverse}(L), 0) \\ (\mathtt{add}(\mathtt{reverse}(L_1), L_2), L \rightarrow x, 0), \ (\mathtt{add}(\mathtt{reverse}(x), L), L \rightarrow \mathtt{take}(L, N), 0) \\ (\mathtt{add}(\mathtt{reverse}(x), \mathtt{take}(L, N)), L \rightarrow x, 0), (\mathtt{add}(\mathtt{reverse}(x), \mathtt{take}(x, N)), \varnothing , -1). \end{array} $$

***Simplified Policy Gradient Estimate for*** $\mathcal {M}_{\phi }$ ***.*** Since our synthesis algorithm will be based on policy gradient, we will now derive a simplified policy gradient for our MDP $\mathcal {M}_\phi$. First, by construction of $\mathcal {M}_{\phi }$, a rollout $\zeta$ has the form

$$\begin{aligned} (P_1,r_1,0),...,(P_m,\varnothing ,q) \end{aligned}$$

where $q=1$ if $P_m \models \phi$ and $q=-1$ otherwise. Thus, the term $\ell (P)$ from Eq. 1 can be simplified as follows:

3 $$\begin{aligned} \ell (P_m)=\sum _{i=1}^{m-1} q \cdot \nabla _{\theta }\log \pi _{\theta }(P_{i},r_i), \end{aligned}$$

where $P_m\sim \mathcal {D}_{\pi _{\theta }}$ is a final state (i.e., complete program or infeasible partial program) sampled using $\pi _{\theta }$. Then, Eq. 1 is equivalently

4 $$\begin{aligned} \nabla _{\theta }J(\pi _{\theta })&\approx \frac{1}{n}\sum _{k=1}^n\ell (P^{(k)}), \end{aligned}$$

where $P^{(k)}\sim \mathcal {D}_{\pi _{\theta }}$ for each $k\in \{1,...,n\}$.

## RL-Based Synthesis Algorithm

In this section, we describe our synthesis algorithm based on reinforcement learning. Our method is an *off-policy* variant of the standard (on-policy) policy gradient algorithm and incorporates additional feedback – in the form of other infeasible programs – provided by the deduction engine when improving its policy parameters. We first give a high-level overview of the synthesis algorithm and then explain how to update the policy.

**Fig. 3.**
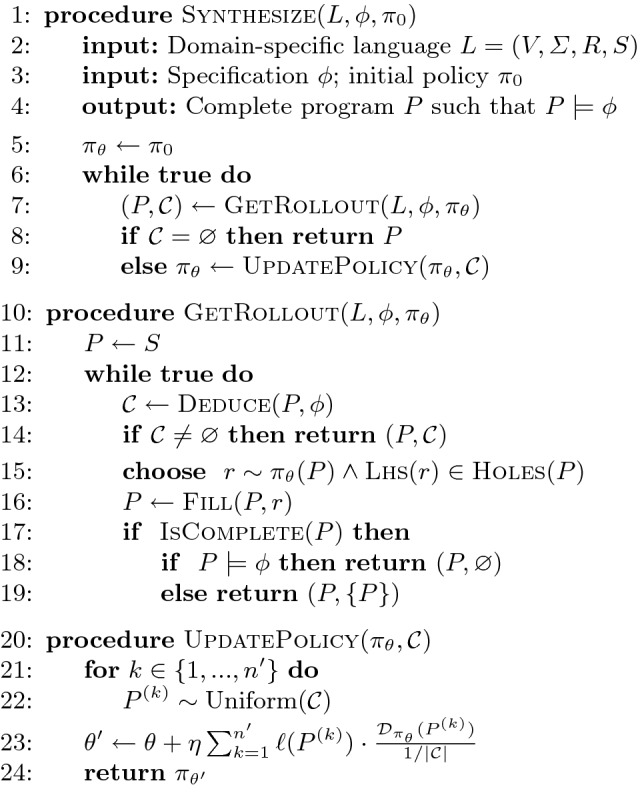
Deduction-guided synthesis algorithm based on reinforcement learning

### Overview of Synthesis Algorithm

Our RL-based synthesis algorithm is presented in Fig. 3. In addition to specification $\phi$ and domain-specific language *L*, this algorithm also takes as input an initial policy $\pi _0$ that has been trained off-line on a representative set of training problems.^1^ In each iteration of the main synthesis loop, we first obtain a rollout of the current policy by calling the GetRollout procedure at line 7. Here, each rollout either corresponds to a complete program *P* or an infeasible partial program. If *P* is complete *and* satisfies the specification, we return it as a solution in line 8. Otherwise, we use feedback $\mathcal {C}$ provided by the deduction engine to improve the current policy (line 9). In the following subsections, we explain the GetRollout and UpdatePolicy procedures in more detail.

### Sampling Rollouts

The GetRollout procedure iteratively expands a partial program, starting from the start symbol *S* of the grammar (line 11). In each iteration (lines 12–19), we first check whether the current partial program *P* is feasible by calling Deduce. If *P* is infeasible (i.e., $\mathcal {C}$ is non-empty), then we have reached a terminal state of the MDP; thus, we return *P* as the final state of the rollout. Otherwise, we continue expanding *P* according to the current policy $\pi _\theta$. Specifically, we first sample an action (i.e., grammar production) *r* that is applicable to the current state (i.e., the left-hand-side of *r* is a hole in *P*), and, then, we expand *P* by calling the Fill procedure (defined in Sect. 3) at line 16. If the resulting program is complete, we have reached a terminal state and return *P*; otherwise, we continue expanding *P* according to the current policy.

### Improving the Policy

As mentioned earlier, our algorithm improves the policy by using the feedback $\mathcal {C}$ provided by the deduction engine. Specifically, consider an infeasible program *P* explored by the synthesis algorithm at line 7. Since Deduce($P,\,\phi$) yields a set of infeasible programs, for every program $P' \in \mathcal {C}$, we know that the reward should be $-1$. As a consequence, we should be able to incorporate the rollout used to construct *P* into the policy gradient estimate based on Eq. (3). However, the challenge to doing so is that Eq. (4) relies on *on-policy* samples – i.e., the programs $P^{(k)}$ in Eq. (4) must be sampled using the current policy $\pi _{\theta }$. Since $P'\in \mathcal {C}$ is not sampled using $\pi _{\theta }$, we cannot directly use it in Eq. (4).

Instead, we use *off-policy* RL to incorporate $P'$ into the estimate of $\nabla _{\theta }J(\pi _{\theta })$ [28]. Essentially, the idea is to use *importance weighting* to incorporate data sampled from a different distribution than $\mathcal {D}_{\pi _{\theta }}$. In particular, suppose we are given a distribution $\tilde{\mathcal {D}}$ over final states. Then, we can derive the following gradient:

5 $$\begin{aligned} \nabla _{\theta }J(\pi _{\theta })&=\mathbb {E}_{P\sim \mathcal {D}_{\pi _{\theta }}}[\ell (P)]\\&=\mathbb {E}_{P\sim \tilde{\mathcal {D}}}\left[ \ell (P)\cdot \frac{\mathcal {D}_{\pi _{\theta }}(P)}{\tilde{\mathcal {D}}(P)}\right] \nonumber \end{aligned}$$

Intuitively, the *importance weight* $\frac{\mathcal {D}_{\pi _{\theta }}(P)}{\tilde{\mathcal {D}}(P)}$ accounts for the fact that *P* is sampled from the “wrong” distribution.

Now, we can use the distribution $\tilde{\mathcal {D}}=\text {Uniform}(\textsc {Deduce}(P', \phi ))$ for a randomly sampled final state $P'\sim \mathcal {D}_{\pi _{\theta }}$. Thus, we have^2^:

#### Theorem 2

The policy gradient is

6 $$\begin{aligned} \nabla _{\theta }J(\pi _{\theta })=\mathbb {E}_{P'\sim \mathcal {D}_{\pi _{\theta }},P\sim \text {Uniform}(\textsc {Deduce}(P', \phi ))}\left[ \ell (P)\cdot \frac{\mathcal {D}_{\pi _{\theta }}(P)}{1/|\textsc {Deduce}(P', \phi )|}\right] . \end{aligned}$$

#### Proof

Note that

$$\begin{aligned} \nabla _{\theta }J(\pi _{\theta })&=\mathbb {E}_{P'\sim \mathcal {D}_{\pi _{\theta }}}[\nabla _{\theta }J(\pi _{\theta })] \nonumber \\&=\mathbb {E}_{P'\sim \mathcal {D}_{\pi _{\theta }},P\sim \text {Uniform}(\textsc {Deduce}(P', \phi ))}\left[ \ell (P)\cdot \frac{\mathcal {D}_{\pi _{\theta }}(P)}{1/|\textsc {Deduce}(P', \phi )|}\right] , \end{aligned}$$

as claimed. $$\square $$

The corresponding estimate of $\nabla _{\theta }J(\pi _{\theta })$ is given by the following equation:

$$\begin{aligned} \nabla _{\theta }J(\theta )\approx \frac{1}{n}\sum _{k=1}^n\frac{1}{n'}\sum _{k'=1}^{n'}\ell (P^{(k,k')})\cdot \frac{\mathcal {D}_{\pi _{\theta }}(P^{(k,k')})}{1/|\textsc {Deduce}(P^{(k)}, \phi )|}, \end{aligned}$$

where $P^{(k)}\sim \tilde{\mathcal {D}}$ and $P^{(k,k')}\sim \text {Uniform}(\textsc {Deduce}(P^{(k)}, \phi ))$ for each $k\in \{1,...,n\}$ and $k'\in \{1,...,n'\}$. Our actual implementation uses $n=1$, in which case this equation can be simplified to the following:

7 $$\begin{aligned} \nabla _{\theta }J(\theta )&\approx \frac{1}{n'}\sum _{k'=1}^{n'}\ell (P)\cdot \frac{\mathcal {D}_{\pi _{\theta }}(P^{(k')})}{1/|\textsc {Deduce}(P, \phi )|}, \end{aligned}$$

where $P\sim \tilde{\mathcal {D}}$ and $P^{(k')}\sim \text {Uniform}(\textsc {Deduce}(P, \phi ))$ for each $k'\in \{1,...,n'\}$.

Now, going back to our synthesis algorithm from Fig. 3, the UpdatePolicy procedure uses Eq. 7 to update the policy parameters $\theta$. Specifically, given a set $\mathcal {C}$ of infeasible partial programs, we first sample $n'$ programs $P^{(1)}, \ldots , P^{(n')}$ from $\mathcal {C}$ uniformly at random (line 22). Then, we use the probability of each $P^{(k)}$ being sampled from the current distribution $\mathcal {D}_{\pi _\theta }$ to update the policy parameters to a new value $\theta '$ according to Eq. 7.

#### Example 5

Suppose that the current policy assigns the following probabilities to these state, action pairs:

$$\begin{aligned} \pi _\theta ( (\mathtt{add}(\mathtt{reverse}(x),L)), L \rightarrow \mathtt{take}(L, N) )&=0.3\\ \pi _\theta ( (\mathtt{add}(\mathtt{reverse}(x),L)), L \rightarrow \mathtt{drop}(L, N) )&=0.3\\ \pi _\theta ( (\mathtt{add}(\mathtt{reverse}(x),L)), L \rightarrow \mathtt{sumUpTo}(L) )&=0.1 \end{aligned}$$

Furthermore, suppose that we sample the following rollout using this policy:

$$ P \equiv \mathtt{add}(\mathtt{reverse}(x), \mathtt{take}(x, N)), $$

This corresponds to an infeasible partial program, and, as in Example 3, Deduce(*P*, $\phi$) yields $\{ P, P'\}$ where $P' \equiv \mathtt{add}(\mathtt{reverse}(x), \mathtt{drop}(x, N))$. Using the gradients derived by Eq. 7, we update the policy parameters $\theta$ to $\theta '$. The updated policy now assigns the following probabilities to the same state, action pairs:

$$\begin{aligned} \pi _{\theta '}( (\mathtt{add}(\mathtt{reverse}(x),L)), L \rightarrow \mathtt{take}(L, N) )&=0.15\\ \pi _{\theta '}( (\mathtt{add}(\mathtt{reverse}(x),L)), L \rightarrow \mathtt{drop}(L, N) )&=0.15\\ \pi _{\theta '}( (\mathtt{add}(\mathtt{reverse}(x),L)), L \rightarrow \mathtt{sumUpTo}(L) )&=0.2 \end{aligned}$$

Observe that the updated policy makes it less likely that we will expand the partial program $\mathtt{add}(\mathtt{reverse}(x),L))$ using the $\mathtt{drop}$ production in addition to the take production. Thus, if we reach the same state $\mathtt{add}(\mathtt{reverse}(x),L)$ during rollout sampling in the next iteration, the policy will make it more likely to explore the sumUpTo production, which does occur in the desired program

$$ \mathtt{add}(\mathtt{reverse}(x), \mathtt{sumUpTo}(x)) $$

that meets the specification from Example 2.

## Implementation

We have implemented the proposed algorithm in a new tool called Concord written in Python. In what follows, we elaborate on various aspects of our implementation.

### Deduction Engine

Concord uses the same deduction engine described by Feng et al. [18]. Specifically, given a partial program *P*, Concord first generates a specification $\varphi$ of *P* by leveraging the abstract semantics of each DSL construct. Then, Concord issues a satisfiability query to the Z3 SMT solver [15] to check whether $\varphi$ is consistent with the provided specification. If it is not, this means that *P* is infeasible, and Concord proceeds to infer other partial programs that are also infeasible for the same reason as *P*. To do so, Concord first obtains an unsatisfiable core $\psi$ for the queried formula, and, for each clause $c_i$ of $\psi$ originating from DSL construct $f_i$, it identifies a set $S_i$ of other DSL constructs whose semantics imply $c_i$. Finally, it generates a set of other infeasible programs by replacing all $f_i$’s in the current program with another construct drawn from its corresponding set $S_i$.

### Policy Network

**Fig. 4.**
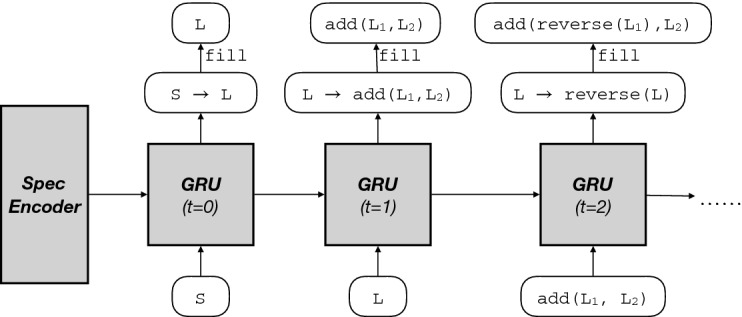
The architecture of the policy network showing how to roll out the partial program in Example 4.

**Architecture.** As shown by Fig. 4, Concord represents its underlying policy using a deep neural network (DNN) $\pi _{\theta }(r\mid P)$, which takes as input the current state (i.e., a partial program *P*) and outputs a probability distribution over actions (i.e., productions *r* in the DSL). We represent each program *P* as a flat sequence of statements and use a recurrent neural network (RNN) architecture, as this is a natural choice for sequence inputs. In particular, our policy network is a gated recurrent unit (GRU) network [13], which is a state-of-the-art RNN architecture. Our policy network has one hidden layer with 256 neurons; this layer is sequentially applied to each statement in the partial program together with the latent vector from processing the previous statement. Once the entire partial program *P* has been encoded into a vector, $\pi _{\theta }$ has a final layer that outputs a distribution over DSL productions *r* based on this vector.

**Pretraining the Initial Policy.** Recall from Sect. 5 that our synthesis algorithm takes a input an *initial policy network* that is updated during the synthesis process. One way to initialize the the policy network would be to use a standard random initialization of the network weights. However, a more effective alternative is to *pretrain* the policy on a benchmark suite of program synthesis problems [44]. Specifically, consider a representative training set $X_{\text {train}}$ of synthesis problems of the form $(\phi , P)$, where $\phi$ is the specification and *P* is the desired program. To obtain an initial policy, we augment our policy network to take as input an encoding of the specification $\phi$ for the current synthesis problem – i.e., it has the form $\pi _{\theta }(r\mid P,\phi )$.^3^ Then, we use supervised learning to train $\pi _{\theta }$ to predict *P* given $\phi$—i.e.,

$$\begin{aligned} \theta ^0={\mathop {\hbox {arg max}}\limits _{\theta }}\sum _{(\phi ,P)\in X_{\text {train}}}\sum _{i=1}^{|P|-1}\pi _{\theta }(r_i\mid P_i,\phi ). \end{aligned}$$

We optimize $\theta$ using stochastic-gradient descent (SGD) on this objective.

Given a new synthesis problem $\phi$, we use $\pi _{\theta ^0}$ as the initial policy. Our RL algorithm then continues to update the parameters starting from $\theta ^0$.

### Input Featurization

As standard, we need a way to featurize the inputs to our policy network – i.e., the statements in each partial program *P*, and the specification $\phi$. Our current implementation assumes that statements are drawn from a finite set and featurizes them by training a different embedding vector for each kind of statement. While our general methodology can be applied to different types specifications, our implementation featurizes the specification under the assumption that it consists of input-output examples and uses the same methodology described by Balog et al. [2].

### Optimizations

Our implementation performs a few optimization over the algorithm presented in Sect. 5. First, since it is possible to sample the same rollout multiple times, our implementation uses a hash map to check whether a rollout has already been explored. Second, in different invocations of the GetRollout procedure from Fig. 3, we may end up querying the feasibility of the same state (i.e., partial program) *many* times. Since checking feasibility requires a potentially-expensive call to the SMT solver, our implementation also memoizes the results of feasibility checks for each state. Finally, similar to Chen et al. [11], we use a 3-model ensemble to alleviate some of the randomness in the synthesis process and return a solution as soon as one of the models in the ensemble finds a correct solution.

## Evaluation

In this section, we describe the results from our experimental evaluation, which is designed to answer the following key research questions:

**Fig. 5.**
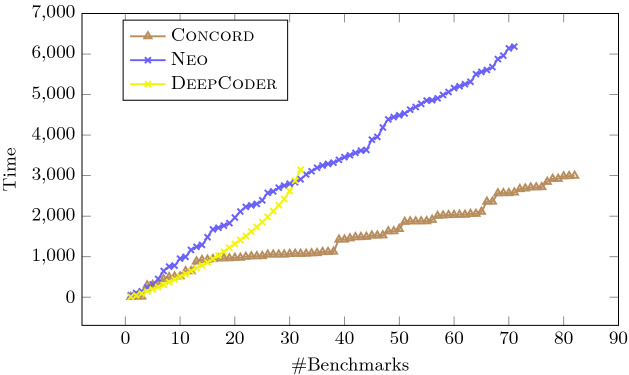
Comparison between Concord, Neo, and DeepCoder

1. How does Concord compare against existing synthesis tools?
2. What is the impact of updating the statistical model during synthesis? (i.e., is reinforcement learning actually useful)?
3. How important is the proposed off-policy RL algorithm compared to standard policy gradient?
4. How important is it to get feedback from the deduction engine when updating the policy?

***Benchmarks.*** We evaluate the proposed technique on a total of 100 synthesis tasks used in prior work [2, 18]. Specifically, these synthesis tasks require performing non-trivial transformations and computations over lists using a functional programming language. Since these benchmarks have been used to evaluate both Neo [18] and DeepCoder [2], they provide a fair ground for comparing our approach against two of the most closely-related techniques. In particular, note that DeepCoder uses a pre-trained deep neural network to guide its search, whereas Neo uses both statistical and logical reasoning (i.e., statistical model to guide search and deduction to prune the search space). However, unlike our proposed approach, neither Neo nor DeepCoder update their statistical model during synthesis time.

***Training.*** Recall that our algorithm utilizes a pre-trained initial policy. To generate the initial policy, we use the same methodology described in DeepCoder [2] and adopted in Neo [18]. Specifically, we randomly generate both programs and inputs, and we obtain the corresponding output by executing the program. Then, we train the DNN model discussed in Sect. 6 on the Google Cloud Platform with a 2.20 GHz Intel Xeon CPU and an NVIDIA Tesla K80 GPU using 16 GB of memory.

### Comparison Against Existing Tools

  To answer our first research question, we compare Concord against both Neo and DeepCoder on the 100 synthesis benchmarks discussed earlier. The result of this comparison is shown in Fig. 5, which plots the number of benchmarks solved within a given time limit for each of the three tools. As we can see from this figure, Concord outperforms DeepCoder and Neo both in terms of synthesis time as well as the number of benchmarks solved within the 5-min time limit. In particular, Concord can solve 82% of these benchmarks with an average running time of 36 s, whereas Neo (resp. DeepCoder) solves 71% (resp. 32%) with an average running time of 99 s (resp. 205 s). Thus, we believe these results answer our first research question in a positive way.

### Ablation Study

To answer our remaining research questions, we perform an ablation study in which we compare Concord against three variants:

- **Concord-noRL:** This variant does not use reinforcement learning to update its policy during synthesis. However, it still uses the pre-trained policy to guide search, and it also uses deduction to prune infeasible partial programs. In other words, Concord-noRL is the same as the synthesis algorithm from Fig. 3 but it does not invoke the UpdatePolicy procedure to improve its policy during synthesis.
- **Concord-NoDeduce:** This variant uses reinforcement learning; however, it does not incorporate feedback from the deduction engine. That is, rather than checking feasibility of partial programs, it instead samples complete programs and uses the percentage of passing input-output examples as the reward signal. Note that this variant of Concord essentially corresponds to the technique proposed by Si et al. [44].^4^
- **Concord-StandardPG:** Recall that our algorithm uses an off-policy variant of the standard policy gradient algorithm to incorporate additional feedback from the deduction engine. To evaluate the benefit of our proposed approach, we created a variant called Concord-StandardPG that uses the standard (i.e., on-policy) policy gradient algorithm. In other words, ConcordStandardPG implements the same synthesis algorithm from Fig. 3 except that it uses Theorem 1 to update $\theta$ instead of Theorem 2.

**Table 1.** Results of ablation study result comparing different variants.

	# solved	Delta to Neo	Avg. time (s)	Speedup over Neo
Concord-noRL	56	−21%	48	1.63$\times$
Concord-NoDeduce	65	−8%	21	3.66$\times$
Concord-StandardPG	65	−8%	27	2.88$\times$
Concord	82	+15%	9	8.71$\times$

The results from this evaluation are summarized in Table 1. Here, the first column labeled “$\#$ solved” shows the number of solved benchmarks, and the second column shows percentage improvement over Neo in terms of benchmarks solved. The third column shows average synthesis time for benchmarks that can be solved by *all* variants and Neo. Finally, the last column shows speed-up in terms of synthesis time compared to Neo.

As we can see from this table, all variants are significantly worse than Concord in terms of the number of benchmarks that can be solved within a 5-min time limit^5^. Furthermore, as we can see from the column labeled “Delta to Neo ”, all of our proposed ideas are important for improving over the state-of-the-art, as Neo outperforms all three variants but not the full Concord system, which solves 15% more benchmarks compared to Neo.

Next, looking at the third column of Table 1, we see that all three variants of Concord are significantly slower compared to Concord in terms of synthesis time. While both Concord and all of its variants outperform Neo in terms of synthesis time (for benchmarks solved by all tools), Concord by far achieves the greatest speed-up over Neo.

In summary, the results from Table 1 highlight that all of our proposed ideas (i.e., (1) improving policy at synthesis time; (2) using feedback from deduction; and (3) off-policy RL) make a significant difference in practice. Thus, we conclude that the ablation study positively answers our last three research questions.

## Related Work

In this section, we survey prior work that is closely related to the techniques proposed in this paper.

***Program Synthesis.*** Over the past decade, there has been significant interest in automatically synthesizing programs from high-level expressions of user intent [2, 6, 21, 23, 25, 39, 40, 46]. Some of these techniques are geared towards computer end-users and therefore utilize informal specifications such as input-output examples [23, 40, 50], natural language [24, 42, 55, 56], or a combination of both [10, 12]. On the other hand, program synthesis techniques geared towards programmers often utilize additional information, such as a program sketch [17, 36, 46, 49] or types [33, 39] in addition to test cases [20, 30] or logical specifications [6, 49]. While the synthesis methodology proposed in this paper can, in principle, be applied to a broad set of specifications, the particular featurization strategy we use in our implementation is tailored towards input-output examples.

***Deduction-Based Pruning.*** In this paper, we build on a line of prior work on using deduction to prune the search space of programs in a DSL [18, 19, 21, 39, 53]. Some of these techniques utilize type-information and type-directed reasoning to detect infeasible partial programs [20–22, 37, 39]. On the other hand, other approaches use some form of lightweight program analysis to prune the search space [18, 19, 53]. Concretely, Blaze uses abstract interpretation to build a compact version space representation capturing the space of all feasible programs [53]; Morpheus [19] and Neo [18] utilize logical specifications of DSL constructs to derive specifications of partial programs and query an SMT solver to check for feasibility; Scythe [50] and Viser [51] use deductive reasoning to compute approximate results of partial programs to check their feasibility. Our approach learns from deduction feedback to improve search efficiency. As mentioned in Sect. 6, the deductive reasoning engine used in our implementation is similar to the latter category; however, it can, in principle, be used in conjunction with other deductive reasoning techniques for pruning the search space.

***Learning from Failed Synthesis Attempts.*** The technique proposed in this paper can utilize feedback from the deduction engine in the form of other infeasible partial programs. This idea is known as *conflict-driven learning* and has been recently adopted from the SAT solving literature [5, 57] to program synthesis [18]. Specifically, Neo uses the unsat core of the program’s specification to derive other infeasible partial programs that share the same root cause of failure, and, as described in Sect. 6, we use the same idea in our implementation of the deduction engine. While we use logical specifications to infer other infeasible programs, there also exist other techniques (e.g., based on testing [54]) to perform this kind of inference.

***Machine Learning for Synthesis.*** This paper is related to a long line of work on using machine learning for program synthesis. Among these techniques, some of them train a machine learning model (typically a deep neural network) to directly predict a full program from the given specification [12, 16, 34, 35]. Many of these approaches are based on sequence-to-sequence models [47], sequence to tree models [56], or graph neural networks [41] commonly used in machine translation.

A different approach, sometimes referred to as *learning to search*, is to train a statistical model that is used to *guide* the search rather than directly predict the target program. For example, DeepCoder [2] uses a deep neural network (DNN) to predict the most promising grammar productions to use for the given input-output examples. Similarly, R3NN [38] and NGDS [26] use DNNs to predict the most promising grammar productions conditioned on both the specification and the current partial program. In addition, there has been work on using concrete program executions on the given input-output examples to guide the DNN [11, 52]. Our technique for pretraining the initial policy network is based on the same ideas as these supervised learning approaches; however, their initial policies do not change during the synthesis algorithm, whereas we continue to update the policy using RL.

While most of the work at the intersection of synthesis and machine learning uses *supervised learning* techniques, recent work has also proposed using reinforcement learning to speed up syntax-guided synthesis [8, 29, 31, 44] These approaches are all on-policy and do not incorporate feedback from a deduction engine. In contrast, in our problem domain, rewards are very sparse in the program space, which makes exploration highly challenging in a on-policy learning setting. Our approach addresses this problem using off-policy RL to incorporate feedback from the deduction engine. Our ablation study results demonstrate that our off-policy RL is able to scale to more complex benchmarks.

***Reinforcement Learning for Formal Methods.*** There has been recent interest in applying reinforcement learning (RL) to solve challenging PL problems where large amounts of labeled training data are too expensive to obtain. For instance, Si et al. use graph-based RL to automatically infer loop invariants [43], Singh et al. use *Q*-learning (a different RL algorithm) to speed up program analysis based on abstract interpretation [45], Dai et al. [14] uses meta-reinforcement learning for test data generation, and Chen et al. [9] uses RL to speed up relational program verification. However, these approaches only use RL offline to pretrain a DNN policy used to guide search. In contrast, we perform reinforcement learning online during synthesis. Bastani et al. has used an RL algorithm called Monte-carlo tree search (MCTS) to guide a specification inference algorithm [3]; however, their setting does not involve any kind of deduction.

## Conclusion and Future Work

  We presented a new program synthesis algorithm based on reinforcement learning. Given an initial policy trained off-line, our method uses this policy to guide its search at synthesis time but also gradually improves this policy using feedback obtained from a deductive reasoning engine. Specifically, we formulated program synthesis as a reinforcement learning problem and proposed a new variant of the *policy gradient* algorithm that is better suited to solve this problem. In addition, we implemented the proposed approach in a new tool called Concord and evaluated it on 100 synthesis tasks taken from prior work. Our evaluation shows that Concord outperforms a state-of-the-art tool by solving 15% more benchmarks with an average speedup of 8.71$\times$. In addition, our ablation study highlights the advantages of our proposed reinforcement learning algorithm.

There are several avenues for future work. First, while our approach is applicable to different DSLs and specifications, our current implementation focuses on input-output examples. Thus, we are interested in extending our implementation to richer types of specifications and evaluating our method in application domains that require such specifications. Another interesting avenue for future work is to integrate our method with other types of deductive reasoning engines. In particular, while our deduction method is based on SMT, it would be interesting to try other methods (e.g., based on types or abstract interpretation) in conjunction with our proposed RL approach.

## Footnotes

1 We explain how to train this initial policy in Sect. 6.

2 Technically, importance weighting requires that the support of $\tilde{\mathcal {D}}$ contains the support of $\mathcal {D}_{\pi _{\theta }}$. We can address this issue by combining $\tilde{\mathcal {D}}$ and $\mathcal {D}_{\pi _{\theta }}$—in particular, take $\tilde{\mathcal {D}}(P)=(1-\epsilon )\cdot \text {Uniform}(\textsc {Deduce}(P',\phi ))(P)+\epsilon \cdot \mathcal {D}_{\pi _{\theta }}(P)$, for any $\epsilon >0$.

3 Including the specification as an input to $\pi _{\theta }$ is unnecessary if we do not use pretraining, since $\phi$ does not change for a single synthesis problem.

4 We reimplement the RL algorithm proposed in [44] since we cannot directly compare against their tool. Specifically, the policy network in their implementation is tailored to their problem domain.

5 To understand the improvement brought by the pre-trainedd policy, we also conduct a baseline experiment by using randomly initialized policy in Concord. Given the setting, Concord can solve as many as 27% of the benchmarks in the given 5-min time limit.
